# Hypercapnic acidosis transiently weakens hypoxic pulmonary vasoconstriction in anesthetized pigs, without affecting the endogenous pulmonary nitric oxide production

**DOI:** 10.1186/cc9601

**Published:** 2011-03-11

**Authors:** MC Nilsson, A Larsson, K Hambraeus-Jonzon

**Affiliations:** 1 Surgical Sciences, Uppsala, Sweden; 2 Karolinska University Hospital, Stockholm, Sweden

## Introduction

Hypercapnic acidosis is often seen in critically ill patients and during protective mechanical ventilation. Conflicting findings regarding the effect of hypercapnic acidosis on endogenous nitric oxide (NO) production and hypoxic pulmonary vasoconstriction (HPV) have been reported. The aim of this study was to test the effects of hypercapnic acidosis on HPV, and the endogenous NO production in hypoxic and hyperoxic lung regions.

## Methods

Sixteen healthy anesthetized pigs were separately ventilated with hypoxic gas to the left lower lobe (LLL) and hyperoxic gas to the rest of the lung. The pigs were then randomized into two groups. Eight pigs received 10% CO_2 _inhalation (Hypercapnia group) to both lung regions, and eight pigs served as the Control group. The NO concentration in exhaled air (ENO), nitric oxide synthase (NOS) activity in lung tissue, and regional pulmonary blood flow were measured.

## Results

There were no significant differences between the Hypercapnia and Control groups for ENO, Ca^2+^-independent, or Ca^2+^-dependent NOS activity in hypoxic or hyperoxic lung regions. The relative perfusion to the hypoxic LLL (QLLL/QT) increased during the first 90 minutes of hypercapnia from 6 (1)% (mean (SD)) to 9 (2)% (*P *< 0.01), and then decreased to the same level as in the Control group where QLLL/QT remained unchanged over time (*P *> 0.05). In addition, hypercapnia increased cardiac output (QT) (*P *< 0.01), resulting in increased oxygen delivery (*P *< 0.01), despite a significant decrease in PaO_2 _(*P *< 0.01).

## Conclusions

Hypercapnic acidosis does not affect the endogenous pulmonary NO production, nor does it potentiate HPV.
